# Association of Serum CXCL13 with Intrarenal Ectopic Lymphoid Tissue Formation in Lupus Nephritis

**DOI:** 10.1155/2016/4832543

**Published:** 2016-11-20

**Authors:** De Ning He, Wen Li Chen, Kang Xia Long, Xiao Zhang, Guang Fu Dong

**Affiliations:** ^1^Department of Rheumatology and Immunology, Pingxiang People's Hospital, Pingxiang, Jiangxi 337055, China; ^2^Department of Infective Diseases, Guangdong General Hospital, Guangdong Academy of Medical Sciences, Guangzhou 510080, China; ^3^Department of Rheumatology and Immunology, Panyu People's Hospital, Guangzhou 510080, China; ^4^Department of Rheumatology and Immunology, Guangdong General Hospital, Guangdong Academy of Medical Sciences, Guangzhou 510080, China

## Abstract

*Aims.* To assess the concentrations of serum CXCL13 and intrarenal ectopic lymphoid tissue (ELT) profiles and their correlation in the patients with lupus nephritis (LN).* Methods.* Serum CXCL13 levels were measured using enzyme-linked immunosorbent assays (ELISA). The expression of CD3, CD20, and CD21 in renal biopsy specimens was tested using immunohistochemical methods.* Results.* Serum CXCL13 levels were significantly higher in the LN group than those in the SLE group without LN and also in the type III and IV LN patients than in type V LN patients. LN patients with positive CD20 expression (CD20+ LN) had a longer disease course and poorer response to combination therapy and higher serum CXCL13 levels than CD20− LN patients. Moreover, the serum CXCL13 level was positively correlated with the number of B cells/HP in the renal tissue of LN patients. The coexpression patterns of CD3, CD20, and CD21 in the renal tissue of LN patients with different WHO pathological types were significantly different. Serum CXCL13 levels were significantly higher in ELT-2 type LN patients than in 0 or 1 type LN patients.* Conclusions.* This study suggested that increased serum levels of CXCL13 might be involved in renal ELT formation and renal impairment process in LN.

## 1. Introduction

Lupus nephritis (LN) is characterized by immune complex deposition and inflammation in glomeruli and the tubulointerstitium, but its precise pathogenesis is not very much clear until now. Many studies have indicated that systemic loss of B-cell tolerance results in the local deposition of immune complexes [1, 2]. With the successful application of targeted B-cell therapy in the treatment of refractory LN, the role of B cells in the pathogenesis of LN has attracted increased consideration. It is known that B lymphocytes are involved in the pathogenesis of systemic lupus erythematosus (SLE) in autoantibody-dependent mechanisms. While some recent studies suggested that autoantibody-independent mechanisms might be more important, the specific mechanism is still unclear [3–5]. Investigations in the MRL/*lpr* mouse model of lupus nephritis have indicated that B cells exert a pathogenic role in the absence of soluble autoantibody production [6]. Local B-cell infiltration and related abnormal expression of ectopic lymphoid tissue (ELT) in the renal tissues of LN mice models was related to the severity of renal impairment [7]. These findings suggested that excessive intrarenal B-cell infiltration and related ELT formation might play an important role in LN occurrence and development. Similar results have been rarely reported in humans.

CXCL13 is a small-molecule cytokine and belongs to the chemokine family, which can regulate transport of various types of white blood cells by binding to its receptor, CXCR5. Because of the ability to attract B cells, it is also known as a B-lymphocyte chemokine (BLC). Research performed in NZB/W-F1 mice and LN patients [4] suggested that CXCL13 played an important role in the initiation and development of LN as a B-lymphocyte chemokine. Whether CXCL13 is related to the renal pathological damage observed in LN and the formation of abnormal B-lymphocyte infiltration-related ELT have not been reported in detail [8, 9].

Thus, we speculated that an abnormally increased serum CXCL13 level could induce excessive chemotaxis of B cells, T cells, and dendritic cells into renal tissues of LN and subsequent excessive ELT formation, which promotes a vicious cycle of the expansion and perpetuation of inflammatory cell aggregates resulting in persistence and chronicity of the renal inflammation in LN patients. This study demonstrated intrarenal B-cell infiltration and related ELT formation in the LN patients with different WHO pathological classifications and showed serum CXCL13 levels pre- and posttreatment and their role in the process of intrarenal ELT formation. Moreover, we explored the role of intrarenal B-cell infiltration and related ELT formation, as well as an increase in serum CXCL13 levels in the pathogenesis, diagnosis, and treatment of LN; CXCL13 may function as a reliable and practical marker of serology and excessive ELT formation as a marker of histology for the clinical diagnosis of LN. They might provide a basis for more precise treatment of LN in the future.

## 2. Patients and Methods

### 2.1. Patients

A prospective study of 114 patients who attended the Department of Rheumatology and Immunology of Guangdong General Hospital was carried out. All patients fulfilled the American College of Rheumatology classification criteria for the diagnosis of SLE [10]. Clinical evidence of LN was obtained in all cases and LN diagnosis was confirmed by examination of renal biopsy specimens. All SLE patients included in this study are with age of no more than 70 years without hepatitis B-associated nephritis or the following complications including infection, a tumor, and severe cardiopulmonary dysfunction.

The following demographic, clinical, and serologic data were collected at the time of renal biopsy: sex, age, duration of SLE and LN, systemic lupus erythematosus disease activity index (SLEDAI), 24 h proteinuria, levels of blood urea nitrogen, serum creatinine, serum C3, C4, antinuclear antibodies (ANA), anti-Sm, anti-ribonucleoprotein (anti-RNP), anti-double-stranded DNA (anti-dsDNA), and antinucleosome antibodies were determined. SLEDAI was used to estimate global disease activity.

This study was performed according to the principles of the Declaration of Helsinki and each participant completed written informed consent before measurements. This study was also approved by the Local Ethics Committee (Institutional Review Board of Guangdong General Hospital).

All LN patients underwent combination therapy with glucocorticoids (GC) and immunosuppressants (IS). IS mainly referred to cyclophosphamide (CTX), mycophenolate mofetil (MMF), and cyclosporine A (CSA). After 6 months of treatment, responses in LN patients were evaluated that included complete remission (CR) and no CR. CR criterion referred to the international standards for evaluating the therapeutic effects in LN [11].

### 2.2. Main Reagents and Instruments

A CXCL13 kit was bought from R&D Company in the United States. Other reagents included Clone PS1, Novacastra as a CD3 antibody (T-cell surface marker); Clone L26, DAKO Company as a CD20 antibody (B-cell surface marker); and Clone 2G9, Gene Company as a CD21 antibody [follicular dendritic cell (FDC) surface marker]. DAKO EnVision plus kit was used for immunohistochemical test. MN1616, German BARD Company, was used as a percutaneous automatic biopsy gun.

### 2.3. Histological Observation of Sampled Renal Specimen

All enrolled clinically diagnosed LN patients undertaken renal biopsy with B-mode ultrasound-guidance. Native kidney biopsies will need to be divided into three parts for conventional light microscopy, electron microscopy, and immunofluorescence studies. Two-thirds of renal biopsy samples were stereotyped in 10% Neutral Buffered Formalin Fixative, dewatered gradually, and soaked in paraffin. Serial sample sections were marked with alternating hematoxylin and eosin (HE), periodic acid-Schiff (PAS), Masson's trichrome, and periodic acid-silver methenamine. One-third of fresh renal tissue was quickly frozen, and 4 *μ*m unfixed and frozen sections were stained with fluorescein isothiocyanate- (FITC-) conjugated rabbit antisera against human IgG, IgA, IgM, C1q, and C3 (DAKO, Denmark), and the direct immunofluorescence of these sections was observed. The pathological diagnosis for biopsy specimens was established using the international standards for pathological classification of LN [12].

### 2.4. Immunohistochemical Staining Examination of CD3, CD20, and CD21 Coexpression in the Kidney

Renal biopsy was performed for all included LN patients. A routine pathological examination was carried out, and CD3, CD20, and CD21 coexpression were detected using immunohistochemical staining kits (DAKO EnVision plus). Serial paraffin-fixed tissue sample sections were cut to a thickness of 3 *μ*m and mounted on positively charged glass slides and kept in a stove at 55°C overnight. Slides were dewaxed and hydrated through a series of washes with xylene and graded alcohols. Endogenous peroxidase was quenched by treatment with 3% H_2_O_2_ for 10 min. Antigen retrieval was carried out by dipping the slides into antigen repair solution (pH 9.0 Tris-EDTA) and heating at 100°C in a microwave at 650 W for 25 min. First mouse monoclonal anti-human CD20, CD3, and CD21 antibodies were added to the slides at dilution of 1 : 800, 1 : 100, and 1 : 200, respectively, in 1% bovine serum albumin in phosphate buffered saline (BSA/PBS) and subsequently stored them 70 min at room temperature. Slides were rinsed with PBS (pH 7.4) between each step (2 times for 5 min). Slides were then mounted with a secondary goat anti-mouse IgG antibody (EnVision/HRP; DAKO) for 40 min at room temperature. Slides were rinsed in PBS (pH 7.4) between each step (2 times for 5 min), then incubated in DAB solution for 1–3 minutes, and rinsed in distilled water. Finally, sections were counterstained with Mayer's hematoxylin, air-dried, cleared in xylene, and coverslipped with mounting medium. Tonsil tissue was stained as a positive control, and primary antibodies were replaced with PBS as a negative control. Positive results manifest with brown particles localized at cytomembrane or cytoplasm of CD3 expression, or cytoplasm of CD20 and CD21 expression.

Based on the CD20, CD3, and CD21 coexpression patterns in the kidney, the LN patients could be divided into 4 categories of ELTs [13]: class 0, class 1, class 2, and class 3. Class 0 had scattered CD3+ cells infiltration only, but without CD20+ cells and CD21+ cells expression; class 1 had scattered CD20+ cells and CD3+ cells infiltration, but without CD21+ cells expression; and class 2 had a nodular aggregation of CD20+ cells and CD3+ cells without CD21+ cells expression. Class 3 demonstrated a nodular aggregation of CD20+ cells and CD3+ cells with CD21+ cells expression.

### 2.5. Determination of Serum CXCL13 Concentration

2 mL venous blood samples were drawn from an enrolled individual in the morning before breakfast and then centrifuged at 3000 r/min for 5 minutes to obtain serum (stored at −80°C until analysis). CXCL13 was tested using an ELISA kit, and quantification was accomplished according to the manufacturer's protocol.

### 2.6. Statistical Analysis

All statistical analyses were performed using the SPSS 13.0 package program. All measurement data with normal distribution are expressed as mean ± standard deviation ($\bar{X}\pm S$) and analyzed by *t*-test between two groups or by ANVOA and SNK test between multiple groups. The measurement data with abnormal distribution such as serum CXCL13, 24-hour urine protein, and anti-dsDNA quantitation are presented as medians (quartile) and were compared using the Mann–Whitney-Wilcoxon test between 2 groups and using nonparametric Kruskal Wallis test among multiple groups. Correlation analyses were performed using the Spearman method. Categorical data are presented as percentages and were compared using Crosstabs and Chi-square test among multiple groups. A *P* value of <0.05 was considered significant.

## 3. Results

### 3.1. Demographic, Clinical Characteristics, and Laboratory Results of LN Patients

Firstly, in this prospective study, 114 SLE patients (89 LN patients and 25 SLE patients without LN) and 21 healthy individuals were enrolled. 89 LN patients (78 women and 11 men; mean age ± SD: 35 ± 12.8 years) were separated into 2 groups according to the renal CD20 expression: LN with intrarenal B cells (LN-B group) and LN without intrarenal B cells (LN non-B group). The LN-B group comprised 69 (77.5%) patients (61 women and 8 men, 30.6 ± 12.2 years) and the LN non-B group comprised 20 (22.5%) patients (17 women and 3 men, 41.5 ± 12.9 years). No significant difference was detected between the two groups in terms of age or gender (*P* > 0.05). When compared with the LN-non-B-cell group, the LN-B-cell group had a longer disease course (21.8 ± 9.9 months versus 9.8 ± 6.2 months, resp.; *t* = 2.185, *P* = 0.045) and a poor complete remission (CR) rate after 6 months of treatment (63.8% versus 90%, resp.; *χ* ^2^ = 2.384, *P* = 0.047). Other clinical characteristics (age, sex, SLEDAI, urine protein quantitation (mg/24 h), serum creatinine, C3, C4, and anti-dsDNA level) between both groups demonstrated no differences. See Table 1.

### 3.2. Histopathology, B-Lymphocyte Infiltration, and ELT Distribution Characteristics in the LN Patients

Of the enrolled 114 SLE patients, 89 LN patients were diagnosed clinically and confirmed by light microscopy, immunofluorescence, and electron microscopy. According to the ISN/RPS classification, 21 patients were diagnosed with type III (23.6%), 53 patients with type IV (59.6%), and 15 patients with type V (16.8%). No type I, II, or VI was found. Lymphocytes infiltration was mainly distributed in the renal interstitium of 77 patients (86.5%), around the renal tubules of 9 patients, and around the glomeruli of 3 patients. Lymphocytes aggregation was regionally distributed in 51 patients (57.3%), while no typical lymph follicles as in tonsil tissue were found. The activity index (AI) score for the renal pathology was 10.7 ± 4.8 (3–19), and the chronicity index (CI) score was 3 ± 1 (1–4). There was no significant difference at the distribution of LN pathological classifications, AI score, and CI score between the LN-B-cell group and the LN-non-B-cell group. See Table 1.

**Figure 1:**
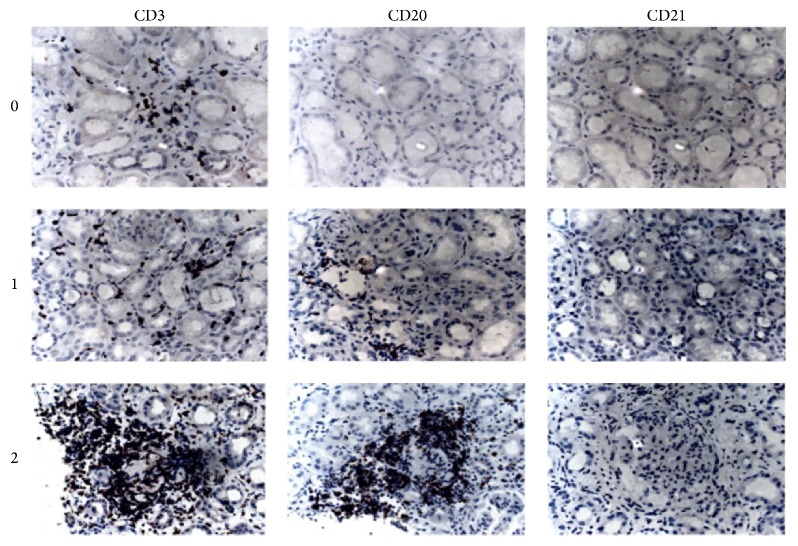
Ectopic lymphoid tissue classifications according to CD20, CD3, and CD21 immunohistochemical features in LN patients (×400). CD20, CD30, and CD21 were stained in serial sections. Class 0 had no CD20+ cell expression, with scattered CD3+ cells only, but without CD21+ cell expression; class 1 had scattered CD20+ cells and CD3+ cells infiltration, but without CD21+ cells infiltration; and class 2 had a nodular aggregation of CD20+ cells and CD3+ cells without CD21+ cell expression.

Based on the CD20, CD3, and CD21 coexpression patterns in the kidney, the 89 LN patients could be divided into 3 categories: class 0, class 1, and class 2. No formation of class 3 ELT was found in the LN patients enrolled in our study. See Figure 1.

The disease duration between the three ELT classes groups was different. The ELT class 2 group showed significantly longer disease duration than the ELT class 0 and the ELT class 1 (*P* = 0.017 and 0.039, resp.), while there was no significant difference (*P* = 0.280) between the ELT class 0 and the ELT class 1. The difference was significant in the AI score between the ELT class 2 and the ELT class 0 (*P* = 0.047), while it was not significant between the ELT class 1 and the ELT class 2 (*P* = 0.056). Other clinical parameters (SLEDAI score, CI score, C3, C4, 24-h urine protein, and anti-dsDNA antibody) in the 3 ELT classes groups demonstrated no differences. See Figure 1 and Table 2.

We noted that the LN kidney tissues for different WHO LN pathological types had different proportions of the three classifications of ELT. See Table 3. The proportions of ELT classes composition in type III, IV, and V LN group were compared using a *χ* ^2^ test, and the differences were significant (*χ* ^2^ = 28.04, *P* < 0.0001). The proportions of ELT classes involving types III and IV were significant (*χ* ^2^ = 10.78, *P* = 0.0046 < 0.0167 [0.05/3]), as were those with types IV and V (*χ* ^2^ = 28.29, *P* < 0.0001 < 0.0167 [0.05/3]). The proportions of ELT classes involving types III and V demonstrated no differences (*χ* ^2^ = 5.07, *P* = 0.0793 > 0.0167 [0.05/3]).

### 3.3. Changes in Serum CXCL13 Levels in LN Patients

**Figure 2:**
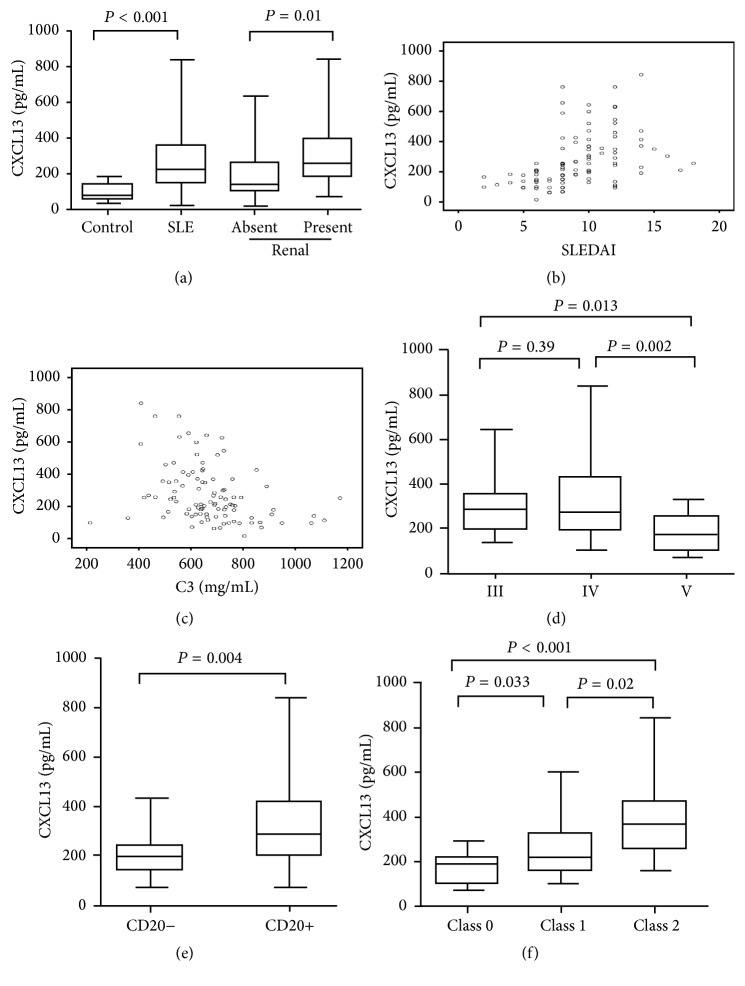
Changes in serum CXCL13 levels in LN patients. CXCL13 levels were increased in LN patients (a). Serum CXCL13 levels correlated with SLEDAI (b) and complement C3 (c). CXCL13 levels were different in LN patients with different WHO pathologic classifications (d), different CD20 expression (e), and different ELT class (f). Horizontal bars indicate median values and the boxes encompass the 25th percentile and 75th percentile, and whiskers indicate maximum and minimum.

According to the presence of renal involvement, SLE patients (*n* = 114) were divided into the LN group (*n* = 89) and the SLE group without renal involvement (SLE-no-LN) (*n* = 25). Serum CXCL13 levels in SLE patients (230.65 [145.92–365.40] pg/mL) were significantly higher than those of healthy individuals (85.13 [62.23–131.58] pg/mL) (*Z* = 4.42, *P* < 0.001). Moreover, serum CXCL13 levels in the LN patients (258.86 [185.50–392.18] pg/mL) were higher than those of the SLE-no-LN group (136.39 [104.23–257.50] pg/mL) (*Z* = 2.53, *P* = 0.01) (Figure 2(a)). After 6 months of treatment, serum CXCL13 levels and SLEDAI were significantly decreased in the LN group (CXCL13: 258.86 [185.50–392.18] pg/mL versus 195.54 [165.98–233.01] pg/mL, resp.; *Z* = −2.59, *P* = 0.009; SLEDAI: 10 [8.75–12] versus 6 [4–6.5], *Z* = −2.56; *P* = 0.01).

After 6 months of immunosuppressive therapy, The LN patients were divided into a complete remission group (*n* = 56) and noncomplete remission group (*n* = 33). Serum CXCL13 levels for the LN group in complete remission (189.46 [114.23–216.50] pg/mL) were significantly lower than those of the LN group with noncomplete remission (286.75 [181.40–386.56] pg/mL) (*Z* = 2.48, *P* = 0.048).

The correlation of serum CXCL13 with SLEDAI in SLE patients was analyzed. Serum CXCL13 levels in SLE patients were positively correlated with SLEDAI (*r* = 0.55, *P* < 0.001) (Figure 2(b)) and negatively correlated with C3 (*r* = −0.39, *P* < 0.001) (Figure 2(c)), and there was no significant correlation with the anti-dsDNA antibody titer (*r* = 0.037, *P* = 0.72).

### 3.4. Serum CXCL13 Levels in LN Patients with Different Pathologic Classifications

Serum CXCL13 levels for type III and IV LN group were higher than those of type V LN group (type III: 287.58 [198.25–355.44] pg/mL; type IV: 271.96 [189.71–434.99] pg/mL; type V: 169.71 [101.09–252.69] pg/mL; type III versus type V, *P* = 0.013; type IV versus type V, *P* = 0.002), while serum CXCL13 levels in type III and IV LN patients demonstrated no difference (*P* = 0.39) (Figure 2(d)).

### 3.5. ELT Patterns in the Renal Tissue of LN Patients and Its Correlation with Changes in Serum CXCL13 Levels

Serum CXCL13 levels in the LN patients with class 2 ELT were significantly higher than those of the LN patients with class 0 and 1 ELT (class 0: 180.96 [101.93–218.88] pg/mL; class 1: 216.91 [158.47–327.15] pg/mL; class 2: 351.80 [251.13–470.36] pg/mL; class 2 versus class 0: *Z* = 4.79, *P* < 0.001; class 2 versus class 1: *Z* = 2.32, *P* = 0.02) (Figure 2(f)). Further, serum CXCL13 levels in the patients with class 1 ELT were higher than those of the patients with class 0 ELT (*Z* = 2.13, *P* = 0.033). Moreover, serum CXCL13 levels were positively correlated with the number of B cells/HP in the renal tissue of LN patients (*r* = 0.41, *P* = 0.048).

## 4. Discussion

The role of long-lived memory plasma cells and B-cell hyperactivity has been recently elucidated in the pathogenesis of SLE [14, 15]. A high prevalence of intrarenal B cells has been noted in immune-mediated diseases, such as renal transplant rejection and glomerulonephritis [16–18] thus indicating that local B-cell infiltrates play a role in tissue injury such as tissue fibrosis, neolymphangiogenesis, and ectopic lymphogenesis [19]. It is hypothesized that intrarenal B cells associated ELTs form part of a local autoimmune system with pivotal involvement in the pathogenesis of lupus nephritis. Our study suggested that the abnormal aggregation of B lymphocytes and related ELT formation in the renal tissue of LN patients might play a unique role in promoting LN persistent progression. Elevated serum CXCL13 level might take part in this process.

In this study, 77.5% of LN patients had excessive B-lymphocyte infiltration in renal tissues. These patients, especially those with focal accumulation of B lymphocytes and T lymphocytes (type 2 ELT), had a significantly longer disease course than those LN patients without B-lymphocyte infiltration. Moreover, LN patients with regional B-cell accumulation had a poor response to combination therapy with GC and IS, suggesting that the abnormal intrarenal B-cell accumulation was related to the longer disease course and poor treatment response in LN. Steinmetz and colleagues [13] observed the renal biopsy specimens of 32 patients with LN and 16 patients with ANCA-associated vasculitis and found that traditional treatment failed to eliminate B-lymphocyte accumulations in the renal tissue of LN patients. In patients with active or refractory LN, more B lymphocytes accumulated in renal tissues and interacted with T lymphocytes and FDC to promote ELT formation, thus leading to chronic renal injury and LN recurrence. Blocking or removing abnormally accumulated B lymphocytes in renal tissues as early as possible might be important to the effective treatment of refractory LN and might be the basis for the therapeutic effect of CD20 monoclonal antibodies in refractory LN [20–22].

This study showed that type 2 ELT was mostly seen in LN patients (*n* = 51, 57.3%), but no typical lymphoid follicles were found. Lymphocyte infiltration was mostly found in the renal interstitium (*n* = 77, 86.5%) that was consistent with a study by Shen et al. [23]. The study enrolled 192 patients with LN and found no CD21+ cell expression in renal tissues. Chang and colleagues [24] conducted a study that enrolled 64 LN patients and found CD20+ B-lymphocyte and CD3+ CD4+ T-lymphocyte infiltration in the renal interstitium and lymphatic germinal center-like structures composed of CD138+ plasma cells and CD21+ dendrite cells. Steinmetz and colleagues [13] observed the renal biopsy specimens of 32 patients with LN and 16 patients with ANCA-associated vasculitis and also found the expression of CD21+ follicular dendrite cells. The diverse ELT patterns demonstrated in various studies may be related to different regions, ethnicities, and inclusion criteria. All the above-mentioned points indicate that preventing ELT formation in the renal tissue of LN patients is an important therapeutic target. Since the renal ELTs in LN patients are obviously different from the secondary lymph node structure such as tonsil, the types of their core immune cell components are different, and their interaction modes may be different, and the roles and abilities of these intrarenal ELTs during LN onset and development may differ from typical secondary lymph tissue, thus necessitating more studies to elucidate.

This study also revealed different ELT distribution characteristics in various LN pathological types. As compared with type V LN, type III and IV LN showed more class 2 ELT distribution. This result is consistent with a study conducted by Shen et al. [23].

Today, the role of CXCL13 in the pathogenesis of SLE has attracted wide attention [25]. We observed changes in serum CXCL13 levels in different WHO classifications of LN and different ELT classes to determine the role of CXCL13 in the pathogenesis of LN and the formation of B-cell-infiltration-related ELT. The results showed that LN patients with WHO types III and IV (especially WHO-IV) and class 1 and class 2 ELT (especially class 2) had significantly higher serum CXCL13 levels. Moreover, serum CXCL13 levels were positively correlated with SLEDAI, the degree of B-cell infiltration in renal tissue, and AI but were negatively correlated with the C3 level, suggesting a possible correlation between abnormally increased serum CXCL13 levels and ELT formation and renal impairment in LN patients. How blood CXCL13 induces locating of B cells mainly in the renal tubular interstitial region and thus forming ELT deserves further study.

In conclusion, this study suggested that abnormal B-cell infiltration and related ELT formation in renal tissues might play an important role in LN occurrence and persistent development, and increased serum CXCL13 levels might take part in ELT formation and the pathological renal impairment process in LN patients. Blocking the biological effects of CXCL13 and the formation of ELT in renal tissues might be important targets in the medical treatment of LN in the future.

## Figures and Tables

**Table 1:** Clinical data of the B-cell group and the non-B-cell group in LN patients.

	LN-B-cell (*n* = 69)	LN-non-B-cell (*n* = 20)	*χ* ^2^/*t*/*H* value	*P* value
Male^*∗*^	8 (11.6)	3 (15.0)	0.786	0.294
Age (years)	30.6 ± 12.2	41.5 ± 12.9	−1.542	0.144
Duration (months)	21.8 ± 9.9	9.8 ± 6.2	2.185	0.045
Urine protein^#^ (mg/24 h)	3 689.5 (1 568.7~8 686.5)	1785.6 (965.8~3 516.5)	−0.679	0.497
Serum Cr (umol/L)	107.2 ± 58.5	98.3 ± 44.9	0.277	0.785
C3 (mg/L)	476.9 ± 261.8	345.8 ± 89.7	0.966	0.350
C4 (mg/L)	151.6 ± 136.7	84.4 ± 29.8	0.956	0.354
Anti-dsDNA^#^ (Iu/mL)	282.5 (115.6~418.6)	312.5 (135.6~462.5)	−0.341	0.733
WHO classification^*∗*^			0.263	0.877
LN (III)	16 (23.2)	5 (25.0)		
LN (IV)	42 (60.9)	11 (55.0)		
LN (V)	11 (15.9)	4 (20.0)		
CI score	3.1 ± 1.6	2.2 ± 1.0	0.559	0.584
AI score	10.3 ± 4.7	10.9 ± 5.1	0.569	0.578
SLEDAI score	17.3 ± 7.9	15.3 ± 3.0	0.082	0.624
CR at 6 months^*∗*^	44 (63.8%)	18 (90%)	2.384	0.047
TR at 6 months^*∗*^	59 (85.5%)	19 (95%)	1.26	0.212

Note: when compared with the LN-non-B-cell group, there was a significant difference only at the disease duration and the CR for 6-month treatment in the LN-B-cell group. CR: complete remission; TR: total remission; dsDNA: double strand DNA; LN: lupus nephritis; AI: activity index; CI: chronicity index; SLEDAI: SLE disease activity index; ^*∗*^expressed in *n* (%); ^#^expressed in median (interquartile range) and nonparameter test.

**Table 2:** The clinical data of patients with different ELT classes of renal tissues.

	Class 0 (*n* = 20)	Class 1 (*n* = 18)	Class 2 (*n* = 51)	*F*/*χ* ^2^ value	*P* value
Age (years)	41.5 ± 12.9	31.4 ± 13.3	30.1 ± 12.4	1.127	0.352
Duration (months)	9.8 ± 6.2	16.4 ± 7.8	24.4 ± 10.2	3.899	0.035
Urine protein (mg/24 h)	1 546.8 (856.9~2 578.6)	2031.6 (956.6~3 956.5)	2365.5 (755.6~9 560.6)	1.123	0.353^*∗*^
Serum creatinine (*μ*mol/L)	98.3 ± 44.9	100.5 ± 42.6	111.4 ± 69.2	0.091	0.914
C3 (mg/L)	345.8 ± 89.7	440.8 ± 278.8	499.5 ± 267.6	0.529	0.600
C4	84.4 ± 29.8	216.9 ± 127.0	110.9 ± 50.8	1.763	0.208
Anti-dsDNA (IU/mL)	196.9 (79.6~796.8)	115.9 (72.6~685.6)	215.6 (115.5~565.7)	0.133	0.936^*∗*^
CI score (points)	5.3 ± 2.2	6.2 ± 1.3	5.6 ± 2.0	0.442	0.652
AI score (points)	5.8 ± 2.6	8.3 ± 4.1	12.5 ± 5.1	2.986	0.041
SLEDAI score (points)	15.3 ± 3.0	14.6 ± 10.0	19.0 ± 6.5	1.707	0.310

Note: ^*∗*^nonparametric test; in all clinical characteristics, there was a significant difference only in the disease duration and the renal pathological activity index (AI) among the class 0, 1, and 2 ELT group.

**Table 3:** ELT formation patterns in renal tissues comprising different LN pathological types.

Pathological types	ELT class		
	Class 0	Class 1	Class 2
Type III (*n* = 21)	7	2	12
Type IV (*n* = 53)	3	14	36
Type V (*n* = 15)	10	2	3

Note: the proportions of ELT classes composition in type III, IV, and V LN group were compared using a *χ* ^2^ test, and the differences were significant (*χ* ^2^ = 28.04, *P* < 0.0001). The proportions of ELT classes involving types III and IV were significant (*χ* ^2^ = 10.78, *P* = 0.0046), as were those with types IV and V (*χ* ^2^ = 28.29, *P* < 0.0001). The proportions of ELT classes involving types III and V demonstrated no differences (*χ* ^2^ = 5.07, *P* = 0.0793).
